# LEAFY, a Pioneer Transcription Factor in Plants: A Mini-Review

**DOI:** 10.3389/fpls.2021.701406

**Published:** 2021-07-05

**Authors:** Nobutoshi Yamaguchi

**Affiliations:** Division of Biological Science, Graduate School of Science and Technology, Nara Institute of Science and Technology, Nara, Japan

**Keywords:** *Arabidopsis thaliana*, chromatin, floral meristem identity, histone, LEAFY, pioneer factor

## Abstract

A subset of eukaryotic transcription factors (TFs) possess the ability to reprogram one cell type into another. Genes important for cellular reprograming are typically located in closed chromatin, which is covered by nucleosomes. Pioneer factors are a special class of TFs that can initially engage their target sites in closed chromatin prior to the engagement with, opening of, or modification of the sites by other factors. Although many pioneer factors are known in animals, a few have been characterized in plants. The TF LEAFY (LFY) acts as a pioneer factor specifying floral fate in *Arabidopsis*. In response to endogenous and environmental cues, plants produce appropriate floral inducers (florigens). During the vegetative phase, LFY is repressed by the TERMINAL FLOWER 1 (TFL1)–FD complex, which functions as a floral inhibitor, or anti-florigen. The florigen FLOWERING LOCUS T (FT) competes with TFL1 to prevent the binding of the FD TF to the *LFY* locus. The resulting FT–FD complex functions as a transient stimulus to activate its targets. Once *LFY* has been transcribed in the appropriate spatiotemporal manner, LFY binds to nucleosomes in closed chromatin regions. Subsequently, LFY opens the chromatin by displacing H1 linker histones and recruiting the SWI/SNF chromatin-remodeling complex. Such local changes permit the binding of other TFs, leading to the expression of the floral meristem identity gene *APETALA1*. This mini-review describes the latest advances in our understanding of the pioneer TF LFY, providing insight into the establishment of gene expression competence through the shaping of the plant epigenetic landscape.

## Introduction

A subset of eukaryotic transcription factors (TFs) possess reprograming activity to change one cell type into another (Meshi and Iwabuchi, 1995; Drouin, 2014). During cell fate reprograming in eukaryotes, TFs control gene expression programs to enable the formation of distinct cell types from the same genome. Different gene expression programs are blocked by chromatin-mediated mechanisms. TF-binding sites are often masked by nucleosomes, which play important roles in genome packaging and gene expression. The nucleosome consists of a segment of DNA wound around two copies of four types of histone proteins. Nucleosome positions in the genome determine the accessibility of the DNA to regulatory proteins.

A special class of TFs called pioneer factors can access their target DNA sequences inside nucleosomes, typically in chromatin regions where the presence of linker histones represses transcription (Iwafuchi-Doi and Zaret, 2014, 2016; Soufi et al., 2015; Iwafuchi-Doi, 2019). The primary functions of pioneer factors are cell fate reprograming and the establishment of competence for changes in cellular fate (Zaret and Carroll, 2011). Notable examples include Sox2 and Oct4, two of the four key TFs that together cause the conversion of mammalian somatic cells into induced pluripotent stem cells (Takahashi and Yamanaka, 2006). Molecular genetic, biochemical, and crystal structural analyses have revealed common features of pioneer factors in animals. A pioneer factor in plants was recently identified by two independent groups (Jin et al., 2021; Lai et al., 2021). In this mini-review, the author describes the latest advances in our understanding of this pioneer factor, LEAFY (LFY).

## Master Regulators are Potential Candidates for Pioneer Transcription Factors

Pioneer TFs are a special group of master regulators. Although not much is known about pioneer TFs in plants, many master regulators have been already identified. Although the definition of the term master regulator or master regulatory gene has expanded since the late 1970s, the original definition was a “gene that occupies the very top of a regulatory hierarchy” which “by its very definition should not be under the regulatory influence of any other gene” (Ohno, 1979). This definition was later modified to describe the hierarchy of cell fate specification in eukaryotes (Nasmyth and Tatchell, 1980; Lewis, 1985; Hamdi et al., 1987; Herskowitz, 1989; Lewis, 1992). Over the next 20 years, the term master regulator was used for genes or proteins with the ability to convert one cell type into another when misexpressed. Classic examples include the myogenic TF MyoD1 in mouse and the hematopoietic TF SCL in zebrafish (Davis et al., 1987; Porcher et al., 1996; Robb et al., 1996; Gering et al., 1998; Tapscott et al., 1998). The basic helix-loop-helix (bHLH) family TF MyoD1 regulates muscle cell differentiation by inducing cell cycle arrest (Olson et al., 2020). Other examples of pioneer factors are the nuclear factor Y (NF-Y) TFs in mouse, and the TFs Oct3/4, Sox2, Klf4, and c-Myc (collectively called the Yamanaka factors) in human and mouse (Takahashi and Yamanaka, 2006; Takahashi et al., 2007; Oldfield et al., 2014). Overall, many animal pioneer factors play key roles in embryogenesis (Lai et al., 2018). Both master regulators and pioneer factors control cell reprograming; therefore, master regulators encoding TFs could be considered candidate pioneer factors. However, the TF families to which most of the animal pioneer factors belong are absent from plants (Lai et al., 2018).

Many TF genes whose activity is sufficient to re-specify cell fate when overexpressed have been identified in *Arabidopsis thaliana*. For example, the master regulator LEAFY COTYLEDON1 (LEC1) is a NF-Y protein that maintains embryonic cell fate during embryogenesis and prevents premature seed germination (West et al., 1994; Lotan et al., 1998; Lee et al., 2003; Tao et al., 2017). Master regulators in plants are involved in cell fate decisions throughout development. For example, the NAC TF VASCULAR-RELATED NAC DOMAIN7 (VND7) promotes xylem vessel cell differentiation (Kubo et al., 2005). Ectopic *VND7* expression was sufficient to confer xylem character. A few bHLH proteins, such as MUTE and FAMA, drive the sequential steps of stomatal differentiation (Ohashi-Ito and Bergmann, 2006; Pillitteri et al., 2007). Overexpression of *FAMA* specified the identity of stomatal and myrosin cells, while *MUTE* misexpression conferred guard cell fate to leaf epidermal cells (Ohashi-Ito and Bergmann, 2006; Pillitteri et al., 2007; Shirakawa et al., 2014). Overexpression of the APETALA 2 (AP2) family TF gene *PLETHORA2* induced ectopic root formation (Aida et al., 2004; Galinha et al., 2007). MADS-box TFs are the core factors involved in floral organ specification (Coen and Meyerowitz, 1991). When overexpressed, they have the ability to transform one type of organ into another (Riechmann and Meyerowitz, 1997). Overexpression of MADS-domain TFs is sufficient to convert leaves into floral organs (Honma and Goto). Among the MADS-box TFs, APETALA1 (AP1) and SEPALLATA3 (SEP3) were proposed to act as pioneer TFs since they can access closed chromatin (Pajoro et al., 2014). The *LFY* gene encodes a plant-specific helix-turn-helix TF (Weigel et al., 1992; Weigel and Nilsson, 1995; Hamès et al., 2008). Although overexpression of *LFY* alone cannot induce ectopic flower formation and does not alter embryogenesis and root formation, overexpression of *LFY* with *WUSCHEL* (encoding a homeodomain TF that promotes stem cell formation) in root tissues conferred floral fate to root cells (Gallois et al., 2004; Wagner et al., 2004). Furthermore, LFY, together with one of its coactivators, the F-box protein UNUSUAL FLORAL ORGANS, can alter leaf development and produce ectopic floral organs (Parcy et al., 1998; Risseeuw et al., 2013).

Among those master regulators, LEC1, AP1, SEP3, and LFY affect chromatin structure (Lai et al., 2018). LEC1 shows sequence similarity to animal pioneer factors. LEC1 might act in the same way as NF-Y in terms of structure and function. MADS-box genes are detected in many eukaryotes, including plants and animals. However, the functional diversification of MADS-domain TFs in plants is much higher than in animals. Indeed, AP1 and SEP3 interact with chromatin remodelers to open chromatin (Smaczniak et al., 2012). On the other hand, MADS-domain TFs in animals act as settler TFs whose genomic binding is principally governed by proximity to open chromatin (Sherwood et al., 2014). Further analysis is required to understand the precise function of the plant MADS domain in the context of chromatin. LFY was the most well-characterized pioneer factor of all the master regulators (Jin et al., 2021; Lai et al., 2021). LFY is only found in plants (Maizel et al., 2005). These results suggested that plant and animal pioneer factors have both the same and different modes of action in terms of structure and function. Further analysis is required to understand the molecular mechanisms of gene expression regulated by plant pioneer factors and its candidates.

## The Basis and Validation of Pioneer Factors

**Figure 1:**
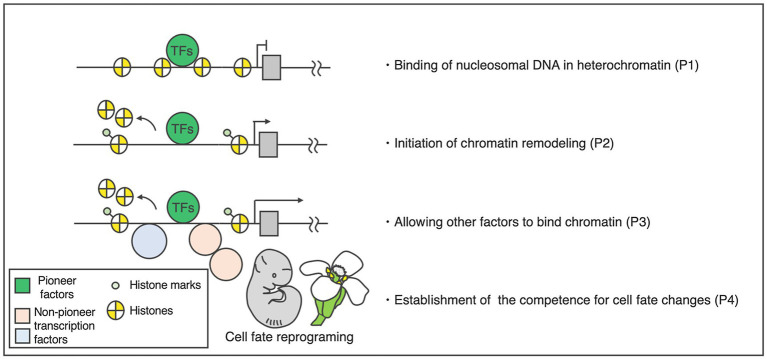
Activity and properties of pioneer factors. Left: hierarchical model of target activation by the pioneer transcription factors. Right: four basic properties of the pioneer factors. The DNA-binding domains of the pioneer factors allow them to target closed chromatin prior to activation [property 1 (P1)]. This binding increases the accessibility of target sites (P2), making the sites accessible to other factors (P3). Pioneer transcription factors play a primary role in cellular programing (P4).

Based on our understanding of animals, pioneer factors are characterized by four major properties (Figure 1; Iwafuchi-Doi and Zaret, 2014, 2016; Soufi et al., 2015; Lai et al., 2018; Iwafuchi-Doi, 2019). The first property is direct binding to a target DNA sequence inside a nucleosome (Figure 1). This feature is often examined through electrophoretic mobility shift assays (EMSAs; Fernandez et al., 2019; Jin et al., 2021; Lai et al., 2021) and sequential TF and core histone chromatin immunoprecipitation (ChIP; Desvoyes et al., 2018; Jin et al., 2021) to provide evidence that putative pioneer factors have specific chromatin-binding properties suitable for such activities. To exclude the possible contribution to other factors, *in vitro* and *in vivo* experiments are required.

The second property is the initiation of chromatin remodeling (Figure 1). To assess this, the chromatin state of a target DNA sequence must be examined before and after the pioneer factors of interest are expressed. Assay for transposase-accessible chromatin with high-throughput sequencing (ATAC-seq), DNase I hypersensitive site sequencing (DNase-seq), or MNase digestion coupled with high-throughput sequencing (MNase-seq) are often used for this purpose (Zhang et al., 2012, 2015; Bajic et al., 2018). ATAC-seq and DNase-seq are used to measure chromatin openness, while MNase-seq is employed to analyze nucleosome occupancy. Prior to the binding of pioneer factors, target DNA sequences are closed and nucleosomal. However, a causal relationship between pioneer factor binding and chromatin opening must be proven as: Merely identifying a correlation between the target DNA sequences of TFs and chromatin opening sites does not necessarily imply cause and is insufficient to validate pioneer factor status.

The third property is allowing for other factors to bind the chromatin (Figure 1). Pioneer factors open up local chromatin regions, thereby directly or indirectly allowing other factors to bind to their targets. Most other TFs cannot initially access a target DNA sequence inside a nucleosome because they lack secondary protein structures important for the recognition of the nucleosome. These non-pioneer TFs are often located alongside the pioneer factor binding sites during or after pioneer factor binding.

The fourth property is the establishment of competence for cell fate changes (Figure 1). This feature is usually analyzed through deletion and ectopic expression of a *TF* gene *in vivo* (Sablowski and Meyerowitz, 1998; Wagner et al., 1999, 2004). The effect of the binding of a TF on DNA accessibility at the target sites inside a nucleosome can then be examined. Since the fourth property of pioneer factors and the definition of master regulators largely overlap, master regulators encoding TFs could be considered candidate pioneer factors. Among the master regulators noted above, only LFY meets all four criteria in plants, so far.

## Regulation of LEAFY Repression and Activation

**Figure 2:**
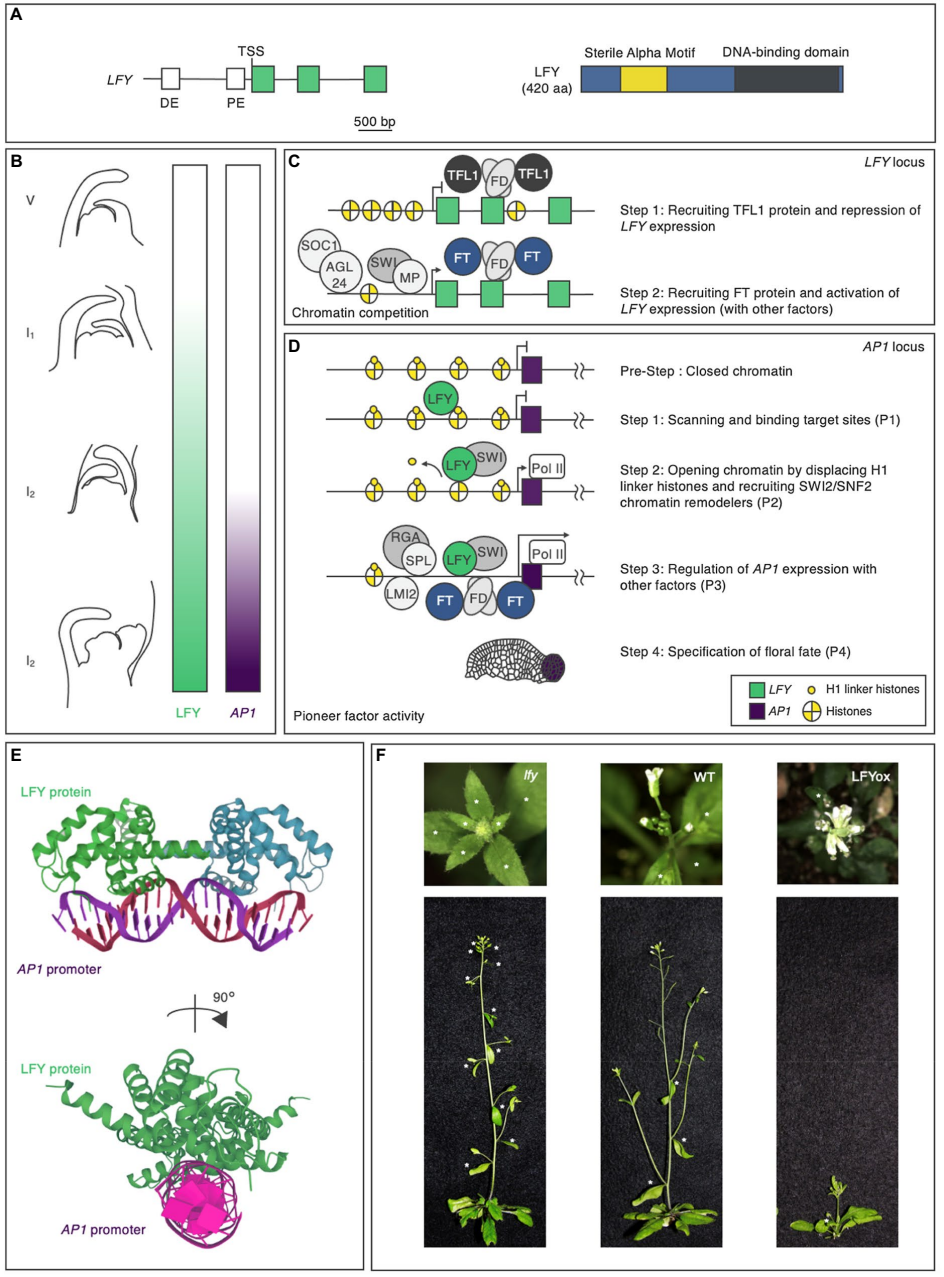
The pioneer transcription factor LEAFY in *Arabidopsis thaliana*. **(A)** *LFY* gene and LFY protein domain structures in *Arabidopsis*. The positions of the conserved regulatory elements (Blázquez and Weigel, 2000) and exons in the gene are indicated in white and green, respectively, and the N-terminal domain, protein-binding domain, and DNA-binding domain in the protein are indicated in blue, yellow, and dark gray, respectively. DE, distal element; PE, proximal element; and TSS, transcription start site. The scale bar represents nucleotide lengths. **(B)** LFY protein accumulation and *AP1* expression during flower formation. Different lateral organs are formed during each phase of the plant lifecycle. During the vegetative (V) phase, rosette leaves form; during the inflorescence phase 1 (I1), cauline leaves and associated secondary inflorescence branches form; and during the inflorescence phase 2 (I2), flowers form. LFY activity and *AP1* expression are indicated by the green and purple color bars, respectively. **(C)** Hierarchical model of *LFY* activation. **(D)** Hierarchical model of *AP1* activation by the pioneer transcription factor LFY. Properties (P1-P4) are shown in Figure 1. **(E)** Crystal structure of LFY and *AP1* double-stranded (ds) DNA. LFY protein is shown in green, while dsDNA is shown in purple. The data were obtained from the Protein Data Bank (https://www.rcsb.org). **(F)** Phenotypes of the *lfy* mutant (left), wild type (middle: WT), and *LFY* overexpressor (right: LFYox). Above: top view. Below: side view. Asterisks indicate secondary inflorescences subtended by cauline leaves on the main stem.

During the vegetative phase, the regulatory region of the *LFY* gene integrates developmental and environmental cues to determine the timing of *LFY* expression (Figures 2A,B; Blázquez et al., 1997, 1998; Blázquez and Weigel, 2000). In addition to the 2.3-kilobase-pair upstream intergenic *LFY* promoter region, which contains distal and proximal elements, the genic region of *LFY* also plays key roles in this integration (Figure 2A; Blázquez et al., 1997, 1998; Blázquez and Weigel, 2000; Yamaguchi et al., 2009, 2013, 2016; Wu et al., 2015; Zhu et al., 2020). The precocious expression of *LFY* in plants during the vegetative phase led to premature flower formation (Weigel and Nilsson, 1995). As a result, these plants produced few seeds. Thus, *LFY* expression must be repressed until a specific time point (Figures 2B,C).

Later work indicated that, to prevent it from specifying floral fate, *LFY* is repressed by the floral inhibitor (anti-florigen) TERMINAL FLOWER 1 (TFL1) during the vegetative phase (Bradley et al., 1997; Ratcliffe et al., 1998; Conti and Bradley, 2007). Consistent with the role of TFL1 in repressing *LFY* expression, *tfl1* mutant, and *TFL1* overexpressor plants showed *LFY* overexpressor and *lfy* mutant phenotypes, respectively, in terms of secondary inflorescence number: The knockout mutation of *TFL1* decreased the number of secondary inflorescences, while the constitutive overexpression of *TFL1* increased the number of secondary inflorescences (Ratcliffe et al., 1998). TFL1 is a member of the phosphatidylethanolamine-binding protein (PEBP) family (Jin et al., 2021) and is thought to act as a transcriptional cofactor in a floral repression complex that includes the basic leucine zipper (bZIP) transcription factor FD (Honma and Goto, 2001; Abe et al., 2005; Ho and Weigel, 2014; Collani et al., 2019; Goretti et al., 2020; Zhu et al., 2020). FD recruits TFL1 to the second exon of *LFY* (Figure 2C; Zhu et al., 2020). This recruitment is largely dependent on FD activity, as TFL1 occupancy is strongly reduced in the null *fd* mutant background (Zhu et al., 2020). The recruitment of TFL1-FD to *LFY* is mediated by evolutionarily conserved bZIP cis-motifs located at the second exon of this gene (Zhu et al., 2020). As is often the case with exonic TF-binding sites, these sites contribute to *LFY* gene expression.

When conditions are right, plants transition from the vegetative to the reproductive phase. In response to endogenous and environmental cues, plants produce the appropriate floral inducers (florigens). FLOWERING LOCUS T (FT), which (like TFL1) belongs to the PEBP family, acts as a major florigen (Kobayashi et al., 1999; Abe et al., 2005; Wigge et al., 2005). The antagonism between FT and TFL1 involves competition for chromatin-bound FD at the *LFY* locus (Zhu et al., 2020). The resulting FT–FD florigen activation complex functions as a transient stimulus at target loci (Figure 2C; Collani et al., 2019; Abe et al., 2019). Other temporal regulators have been identified as *LFY* activators, such as SUPPRESSOR OF CONSTANS OVEREXPRESSION 1, AGAMOUS-LIKE24, SQUAMOSA PROMOTER-BINDING PROTEIN-LIKE3, and MYB33 (Figure 2C; Gocal et al., 2001; Liu et al., 2008; Yamaguchi et al., 2009). Whether these activators interact with each other to determine the timing of *LFY* expression in the context of chromatin is unknown.

The timing and the location of *LFY* expression must be specified to confer floral fate on specific cells. The IAA-AUXIN RESPONSE FACTOR (ARF) module and AP2-type TFs control *LFY* expression in floral primordia, as indicated by their similar expression patterns (Karim et al., 2009; Yamaguchi et al., 2013, 2016; Wu et al., 2015). ARF5/MONOPTEROS (MP) and two SWI-SNF ATPase chromatin remodeling factors, SPLAYED (SYD) and BRAHMA (BRM), activate shared targets including *LFY* (Figure 2C; Bezhani et al., 2007; Yamaguchi et al., 2013; Wu et al., 2015). The MP-SYD/BRM complex associates with evolutionarily conserved and biologically important auxin response elements (AuxREs) located in the proximal region of the upstream intergenic *LFY* promoter (Yamaguchi et al., 2013; Boer et al., 2014; Wu et al., 2015). The MP-SYD/BRM complex unlocks chromatin and allows shared target loci of AuxREs to become accessible. Numerous genes encoding AP2-type TFs, such as *AINTEGUMENTA* (*ANT*), *AINTEGUMENTA-LIKE 6*/*PLETHORA 3*, *PUCHI*, *DÖRNROSCHEN* (*DRN*), and *DÖRNROSCHEN-LIKE* (*DRNL*), show expression patterns overlapping with that of *LFY* in floral primordia (Nole-Wilson et al., 2005; Karim et al., 2009; Krizek, 2009; Yamaguchi et al., 2013; Chandler and Werr, 2017). In higher-order or sensitized mutants of these genes, *LFY* expression in floral primordia is reduced, pointing to their roles in upregulating *LFY* expression (Yamaguchi et al., 2016). Among these TFs, ANT, and AIL6 moderately bind to the upstream intergenic *LFY* promoter region near the proximal region (Yamaguchi et al., 2016). However, how PUCHI, DRN, and DRNL contribute to the activation of *LFY* expression remains to be clarified.

## Initial Targeting of the Pioneer Factor LEAFY and Subsequent Events

Once *LFY* is transcribed in the correct spatiotemporal manner through the actions of the TFs described above, LFY influences fate specification *via* transcriptional regulation, functioning as a pioneer factor. The regulatory network downstream of LFY comprises a set of interlocking feed-forward loops that control the timing of the upregulation of *AP1*, encoding a TF that specifies floral fate (Mandel et al., 1992; Parcy et al., 1998; Wagner et al., 1999; William et al., 2004; Benlloch et al., 2011; Moyroud et al., 2011; Winter et al., 2011; Sayou et al., 2016). LFY specifies not only floral fate, but also flower primordium founder fate and floral organ fate. In this review, the author does not discuss functions other than floral fate specification, since they are covered in detail in recent reviews (Ó’Maoiléidigh, 2014; Wang and Jiao, 2018). LFY meets all four properties for *AP1* regulation (Figure 2D).

LFY is composed of two domains, a sterile alpha motif (SAM) oligomerization N-terminal domain and a C-terminal DNA-binding domain (DBD), a helix-turn-helix fold that by itself dimerizes on DNA (Figure 2A). Although the SAM oligomerization domain itself does not affect DNA binding *in vitro*, it is required for accessing regions with low-affinity-binding sites and closed chromatin (Sayou et al., 2016). LFY recognizes semi-palindromic 19-bp *cis*-elements through its DBD (Figure 2E; Hamès et al., 2008; Moyroud et al., 2011; Winter et al., 2011; Sayou et al., 2016). Besides *cis*-elements in the DNA targets themselves, *in vivo* modifications in the context of chromatin are important for the DNA-binding activity of LFY. EMSA data indicate that LFY associates with the nucleosomal regulatory region of *AP1 in vitro* (Jin et al., 2021; Lai et al., 2021; first property). When the *cis*-element was mutated, LFY did not bind to the nucleosomal substrate, suggesting that LFY binds to nucleosomal DNA *via* its *cis*-element *in vitro* (Jin et al., 2021). MNase-seq and sequential ChIP results supported the notion that LFY binds to the nucleosomal regulatory region of *AP1 in vivo* (Jin et al., 2021).

A LFY-binding test using DAP-seq and ampDAP-seq revealed that LFY is able to bind to both methylated and non-methylated DNA. Whereas an increased number of methylated cytosines in the whole bound region strongly decreases the binding for the two methylation-sensitive TFs (such as ERF018) in DAP relative to ampDAP, LFY binding was only mildly affected (O'Malley et al., 2016; Bartlett et al., 2017; Lai et al., 2021). Since DNA methylation is often seen in closed chromatin regions (Yin et al., 2017; Klemm et al., 2019), pioneer factors may have to access DNA regardless of DNA methylation status. Based on structural analysis of the DBD of LFY in complex with *AP1*, hydrophobic contacts between LFY and DNA could be enhanced by the presence of a methyl group (Hamès et al., 2008). The role of DNA methylation in LFY binding needs to be clarified in the future.

Initial chromatin opening by LFY is mediated by the displacement of the histone H1 linker and the recruitment of SWI/SNF chromatin remodelers (second property). The structural similarity was observed between the helix-turn-helix DBD of LFY and linker histone H1. H1-deficient plants show pleiotropic defects during cell fate specification (Hamès et al., 2008; Rutowicz et al., 2019; Jin et al., 2021). After LFY induction, LFY removes the H1 linker at the *AP1* locus (Jin et al., 2021). LFY interacts with SYD and BRM to open up chromatin by remodeling the nucleosomes at regulatory regions (Bezhani et al., 2007; Wu et al., 2012). SWI3B, a core component of both SYD and BRM, is recruited after LFY induction (Jin et al., 2021). Furthermore, the induction or constitutive expression of *LFY* increases local chromatin accessibility, as revealed by formaldehyde-assisted identification of regulatory elements (FAIRE) analysis (Jin et al., 2021; Lai et al., 2021).

After opening up local chromatin, LFY directly or indirectly allows other TFs to bind their targets (Pastore et al., 2011; Yamaguchi et al., 2014; Jin et al., 2021; third property). In addition to directly activating *AP1*, LFY promotes the activation of regulators of *AP1*. *LATE MERISTEM IDENTITY* (*LMI2*), encoding an MYB TF, is a direct target of LFY. Like LFY, LMI2 also directly promotes *AP1* expression (Pastore et al., 2011). The LFY-binding motif and the LMI2-binding motif in the *AP1* regulatory region are in close proximity (Pastore et al., 2011; Jin et al., 2021). The simultaneous activation of LFY and LMI2 revealed that LMI2 binding in the context of the nucleosome requires the pioneer function of LFY (Jin et al., 2021). LFY also activates the expression of *EUI-LIKE P450 A1* (*ELA1*), which encodes a gibberellin-inactivating enzyme; increased LFY activity leads to reduced gibberellin levels and increased DELLA protein levels (Yamaguchi et al., 2014). A DELLA transcriptional cofactor interacts with the TF SPL9 at the regulatory regions of *AP1* (Yu et al., 2012; Yamaguchi et al., 2014) and activates *AP1* in parallel with LFY. Coherent dual feed-forward loops induce *AP1* expression.

LFY has the ability to convert cell fate when overexpressed (Weigel and Nilsson, 1995; Wagner et al., 1999, 2004; fourth property). Loss or reduction of LFY activity resulted in an increased number of secondary inflorescences, whereas constitutive overexpression of *LFY* caused precocious flower formation without secondary inflorescences (Figure 2F; Weigel et al., 1992; Weigel and Nilsson, 1995). LFY conferred floral fate to root explant cells and allowed callus to form flowers and floral organs without producing leaves (Wagner et al., 2004). Regardless of the tissue, LFY alters gene expression programs *via* the same chromatin-mediated mechanisms. Not only in floral cells, but also in root explant cells, the interaction between LFY and nucleosomes, displacement of the histone H1 linker, and recruitment of SWI/SNF chromatin remodelers increased chromatin accessibility, leading to upregulation of *AP1* (Jin et al., 2021; Lai et al., 2021). Since root explants lack floral factors, and the root explants did not previously exhibit floral fate, this indicates that LFY alone is sufficient to trigger cellular reprograming to determine floral fate.

## Conclusion and Future Prospects

During cell fate specification in eukaryotes, cellular reprograming is controlled by pioneer TFs. In the past three decades, research into the roles of plant TFs in cell fate specification using phenotypic, transcriptome, epigenome, and crystal structure analyses has revealed the importance of TFs whose misexpression changes the fate of one cell type into another. Two independent groups recently uncovered the initial targeting mechanism by which LFY can engage closed chromatin. This initial targeting of nucleosomal DNA allows LFY to initiate reprograming of silent genes, leading to cell type conversion. Although LFY regulates a lot of downstream target genes involved in flower primordium founder cell fate, and floral organ fate as well, whether LFY also has the potential to function as a pioneer factor in the context of other target genes is not yet known. LFY regulates *AP1* expression as a pioneer factor, but how different target genes are regulated by LFY in a spatiotemporal manner needs further study. There may be differences in the DNA-binding specificity of LFY in acting as a pioneer factor *vs*. a non-pioneer factor.

One major limitation to current research on pioneer factors in plants is the lack of a general understanding of these factors; additional pioneer factors in plants need to be identified to provide more data about this class of TFs. Although only LFY fulfills all four criteria of pioneer factors, certainly others will eventually be identified. Although linker histone H1 and SWI/SNF chromatin remodelers play key roles in allowing LFY to exert its roles as a pioneer factor, the initial targeting mechanisms for each pioneer factor could be different. Interestingly, H1- and SWI/SNF-deficient plants show pleiotropic defects, affecting diverse processes including seed dormancy, lateral root formation, root hair fate, stomate formation, and callus formation. There may be pioneer factors that control these developmental processes *via* a mechanism shared with LFY. Various approaches will also be useful for identifying pioneer factors that engage their target sites in chromatin *via* unique mechanisms. Detailed studies of diverse TFs will likely reveal subsets of factors with dominant nucleosome-binding function and pioneer activity in plants. Further understanding of how pioneer factors function will lay the foundation for developing methods to manipulate cell fate in plants.

## Author Contributions

NY: conceptualization, funding acquisition, and writing.

### Conflict of Interest

The author declares that the research was conducted in the absence of any commercial or financial relationships that could be construed as a potential conflict of interest.
